# Grp94 in complexes with IgG is a soluble diagnostic marker of gastrointestinal tumors and displays immune-stimulating activity on peripheral blood immune cells

**DOI:** 10.18632/oncotarget.12141

**Published:** 2016-09-20

**Authors:** Elisa Tramentozzi, Erlis Ruli, Imerio Angriman, Romeo Bardini, Michela Campora, Vincenza Guzzardo, Rita Zamarchi, Elisabetta Rossi, Massimo Rugge, Paola Finotti

**Affiliations:** ^1^ Department of Biology, University of Padua, 35121 Padua, Italy; ^2^ Department of Statistical Sciences, University of Padua, 35121 Padua, Italy; ^3^ Division of General Surgery, University of Padua, 35121 Padua, Italy; ^4^ Department of Surgical and Diagnostic Sciences (DISC), University of Genova, 16132 Genova, Italy; ^5^ Istituto Oncologico Veneto IOV, I.R.C.C.S., 35128 Padua, Italy; ^6^ Department of Surgery, Oncology and Gastroenterology, Oncology Section, University of Padova, Padova, Italy & IOV-IRCCS, Padova, Italy; ^7^ Department of Medicine, DMED, University of Padua, 35121 Padua, Italy; ^8^ Department of Pharmaceutical and Pharmacological Sciences, University of Padua, 35131 Padua, Italy

**Keywords:** gastrointestinal neoplasms, heat shock proteins, immunoglobulins, immunomodulation, biomarkers

## Abstract

Glucose-regulated protein94 (Grp94), the most represented endoplasmic reticulum (ER)-resident heat shock protein (HSP), is a tumor antigen shared by different types of solid and hematological tumors. The tumor-specific feature of Grp94 is its translocation from the ER to the cell surface where it displays pro-oncogenic functions. This un-physiological location has important implications for both the tumor pathology and anti-tumor therapy. We wanted to address the question of whether Grp94 could be measured as liquid marker in cancer patients in order to make predictions of diagnostic and therapeutic relevance for the tumor. To this aim, we performed an in-depth investigation on patients with primary tumors of the gastrointestinal (GI) tract, using different methodological approaches to detect Grp94 in tumor tissues, plasma and peripheral blood mononuclear cells (PBMCs). Results indicate that Grp94 is not only the antigen highly expressed in any tumor tissue and in cells of tumor infiltrates, mostly B lymphocytes, but it is also found in the circulation. However, the only form in which Grp94 was detected in the plasma of any patients and in B lymphocytes induced to proliferate, was that of stable complexes with Immunoglobulin (Ig)G. Using a specific immune-enzyme assay to measure plasma Grp94-IgG complexes, we showed that Grp94-IgG complexes were significantly increased in cancer patients compared to healthy control subjects, serving as diagnostic tumor biomarker. Results also demonstrate that the stimulation of patient PBMCs with Grp94-IgG complexes led to an increased secretion of inflammatory cytokines that might drive a potentially beneficial anti-tumor effect.

## INTRODUCTION

A lot of experimental evidence has so far been accumulated to show that Grp94, the most abundant ER-resident HSP, plays a fundamental role in the pathogenesis of different types of tumors, both solid and hematological [1–7]. The ER stress and the increased metabolic demand associated with the intense, uncontrolled cell proliferation are a potent stimulus for inducing the expression of HSPs, mostly those residing in the ER, deputed to maintain the cellular homeostasis [8]. As a molecular chaperone, Grp94 is involved in the process of protein quality control of a specific and limited set of proteins [9], including the maturation and the correct assembly of IgG [10]. However, besides chaperoning cellular proteins, a property also shared with other HSPs, Grp94 has a peculiar, important role in modulating the activity of the immune system since it assists the MHC class I molecules in the antigen presentation, thus inducing the maturation and activation of various cells involved in both the innate and adaptive immune response [11]. In performing this specific function, Grp94 loses its ER-retention sequence and trans-locates to the cell membrane where it assumes different functions [12–14]; in particular, it behaves as a potent cytokine and controls, among others, the maturation of proteins involved in cell proliferation, apoptosis and inflammation, modulating the activity of specific cellular signaling pathways [8].

Cell-surface Grp94 has been taken as specific indicator of tumor malignancy, more reliable than other ER Grps and HSP90 in predicting the aggressiveness and invasiveness of the tumor itself [15, 16]. Correlation between over-expression of Grp94 and clinic-pathology of the tumor has been described especially in tumors of the GI tract [4, 14, 17–19], but also in non-small-cell lung carcinoma [15] and in hematological tumors [6]. Since Grp94 is crucially involved in the regulation of multiple steps of tumorigenesis and is often induced in tumors that have developed resistance to the conventional chemotherapy [8, 20], it has become an attractive target for both drugs and vaccine development to combat tumor growth and recurrence [12, 21, 22]. The fact that the expression on cell surface and even the secretion of Grp94 can only occur in malignant but not normal cells, offers the unique opportunity to specifically target the protein to halt the tumor progression in a much safer manner compared to the conventional anti-cancer therapy. At the same time, identification of Grp94 as tumor antigen renders Grp94 a protein suitable for developing specific anti-cancer therapeutic vaccines [20, 23, 24]. However, in order to progress in any of the Grp94-specific anti-tumor therapies it is mandatory to obtain preliminary information about the level of the Grp94 expression that only can help predict the therapeutic success [25]. Although an increasing number of scientific papers points to Grp94 as the most diffuse tumor biomarker and reliable index of the malignancy and negative prognosis of the tumor, no one has so far addressed the question of how to measure the protein in plasma or other biological fluids, the condition that could confer on Grp94 the validity of a tumor biomarker of clinical utility. The difficulty in performing such measurements depends on the particular structural and functional properties displayed by Grp94 when exposed in the extra-cellular environment, especially in plasma where it is found only in stable complexes with IgG [26–28]. Indeed, it has been observed that when exposed outside the cell, Grp94 rapidly binds to IgG through non-immune, stable binding at specific binding sites [29, 30]. Complexes that thus form acquire a long life span and display important immune-inflammatory properties [26, 29].

We wanted to handle the issue of how Grp94 can spread from cancer cells, where it is expressed as tumor antigen, into the circulation, becoming a measurable diagnostic and prognostic tumor biomarker whose expression might also be exploited to predict an immune-modulating activity of therapeutic utility. To this aim, we performed a thorough investigation on patients with different tumors of the GI tract, to detect and measure Grp94 expression in both tumor specimens and blood, also testing the response that immune circulating cells of patients might have under the challenge with Grp94. Our results indicate that Grp94 is liberated from cancer cells into plasma where it can be measured in form of complexes with IgG whose expression is significantly increased in cancer patients with respect to the normal counterpart. Results also reveal that Grp94 in complex with IgG can drive an inflammatory response in circulating immune cells consistent with a potentially beneficial anti-tumor effect.

## RESULTS

### Grp94 is an antigen shared by any tumor of the gastrointestinal tract

**Figure 1:**
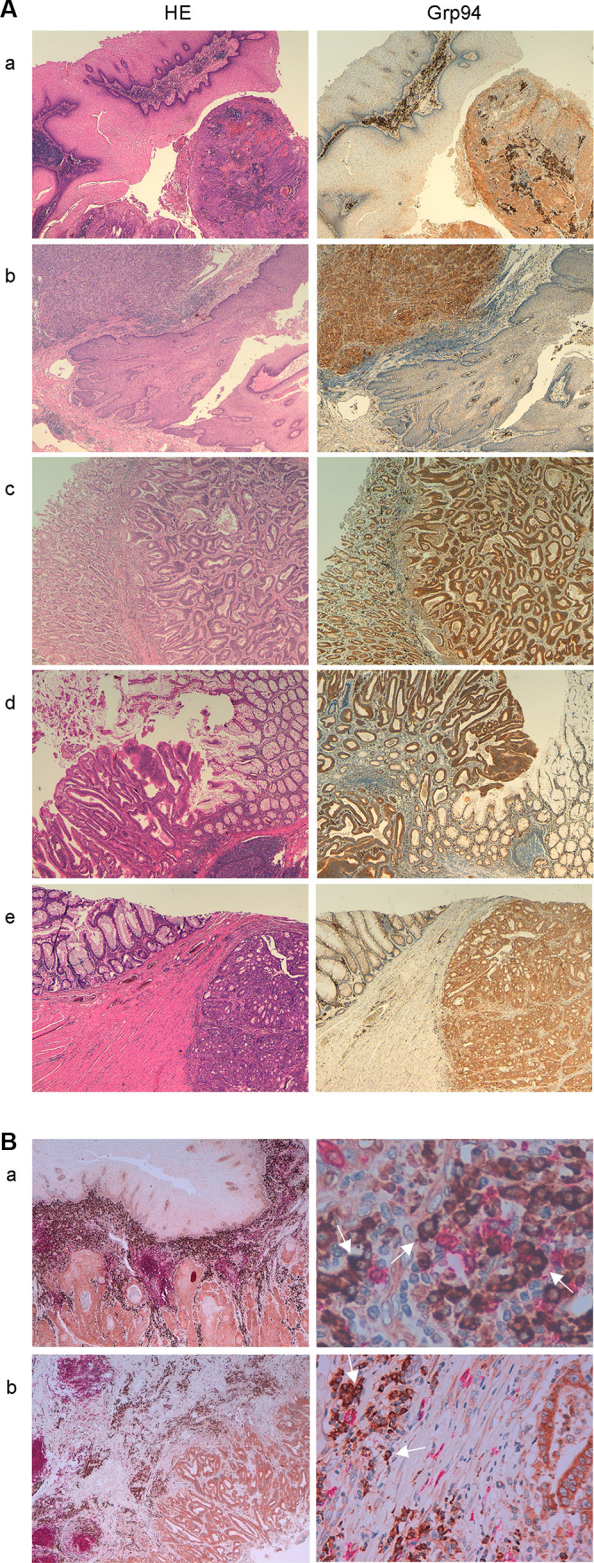
Grp94 marks cancer cells of any tumor of the GI tract and cells of tumor infiltrates (**A**) Specimens of different tumors (a, oesophageal squamous cell carcinoma; b, adeno-carcinoma of the gastro-enteric junction; c, tubular-type adeno-carcinoma of the stomach; d and e, large bowel adeno-carcinomas) were stained with H&E and incubated with rat monoclonal anti-Grp94 Abs for specific immunostaining, as specified in Methods. Magnifications are 10× (a–d) and 20× (e). (**B**) Double immunostaining for both CD20^+^ cells (anti-human CD20cy Abs) and Grp94 of specimens of: a, oesophageal squamous cell carcinoma (20×, left) showing diffuse infiltration of B cells into the tumor stroma with the enlargement (63×, right) showing that the Grp94-positive cells are mostly plasma cells (arrows); b, large bowel carcinoma (10×, left) with enlargement (40×, right) revealing the same features as in a.

The characteristics of patients and tumors are listed in Table 1. No significant differences were found between males and females for age, stage and grade of tumors (Table 1). The majority of patients (70%), irrespective of gender, had adenocarcinomas of the large bowel, characterized by a prevalent mucinous component and a variable extension to pericolic fat. Gastric tumors were both tubular-type adenocarcinomas and signet ring cell carcinomas, whereas tumors of oesophagus and of the gastro-oesophageal junction were a squamous cell carcinoma and an adenocarcinoma, respectively. To see whether Grp94 was the tumor antigen shared by any patient irrespective of the anatomical site and stage of the tumor, we performed histo-immunochemistry (IHC) analysis on any sample of tumor tissue (and its normal counterpart), and measured Grp94 in patients’ plasma. IHC revealed an intense positivity for Grp94 in the tumor tissue of any patient (Figure 1A and Supplementary Figure S1) with the exception of one single patient (female, with G2 adenocarcinoma of rectum) whose tumor infiltrate however stained positively for Grp94 (Supplementary Figure S1). Apparently, Grp94 staining did not show any significant association with both tumor stage (*p* = 0.39) and grading (*p* = 0.45), although tumors at later stages (*p* = 0.0074), but not of higher grade (*p* = 0.34) showed a stronger expression of Grp94 (Table 2). At variance with what observed in the normal counterpart of any tissue sample, in which the expression of constitutive Grp94 was inconstantly and weakly evidenced in the cell, in tumor tissue Grp94 was also localized on the apical part of the cell (Supplementary Figure S1) and was also found in the extracellular secretion. This was in keeping with previous results showing that under inflammatory stimuli, including the neoplastic transformation, Grp94 trans-locates from ER to the cell membrane acquiring the function to sustain the growth and the diffusion of tumor [12, 14]. Interestingly also, in any tumor tissue, irrespective of the histological type and anatomical site, a diffuse cellular infiltrate was apparent that intensely stained for Grp94 (Figure 1 and Supplementary Figure S1), suggesting the involvement of the lymphocyte population in taking up and spreading the antigen protein. To investigate this aspect further, we found that cells of tumor infiltrates were for the most part represented by B lymphocytes, as evidenced by staining with anti-CD20^+^ Abs (Figure 1B), and that in a double immune-staining for both Grp94 and CD20^+^, the cells positive for Grp94 were mostly plasma cells, easily identified for their typical morphological aspect (Figure 1B, arrows in right panels of higher enlargement).

**Table 1:** Characteristics of patients and tumors

	Patients (*n*= 27)	Males* (*n*= 13)	Females* (*n* = 14)
Mean age (yr) (range)	66 (40–94)	67 (40–94)	64 (49 – 80)
Tumor site			
oesophagus/GEJ	2	1	1
stomach	6	4	2
colon	8	3	5
sigma/rectum	11	5	6
Tumor stage			
I/II	10	5	5
III/IV	17	8	9
Tumor grading			
G2	13	5	8
G3	14	8	6
Main laboratory findings			
^§^reduction in RBC count	13/27	9	4
^¶^coagulation alterations	15/26	10	5
^≠^plasma protein alterations	17/23	7	10

Stage I/II includes T2–T3 tumors of the TNM classification (no regional lymph nodes involved, no metastasis). Stage III/IV includes T2–T4 tumors (one or more lymph nodes involved, either with or without metastasis). Sites of stage I/II tumors: oesophagus (1), stomach (1), colon (3) and sigma/rectum (5); sites of stage III/IV tumors: GEJ (gastro-oesophageal junction) (1), stomach (5), colon (5) and sigma/rectum (6).

Sites of G2 tumors: oesophagus (1), stomach (2), colon (3) and sigma/rectum (7). Sites of the G3 tumors: GEJ (1), stomach (5), colon (4) and sigma/rectum (4).

* Males did not differ from females for age (Mann-Whitney test, *p* = 0.56), nor for tumors site, stage and grade (Fisher's exact test, *p* = 0.81, *p* = 1.0 and *p* = 0.45, respectively)

§ Associated reductions in Hb concentration and Ht

¶ Reductions of PT and A-APTT below the lower limit

≠ Reduction in HSA and γ-globulin concentrations, increases in α1 and α2 globulins

Of six patients tested for serum tumor biomarkers (S-CA 19-9, S-CA 15-3, S-CEA, S-CA 125, AFP) at the admission, only one was found positive for CEA (male patient, tumor of sigma, stage I/II).

**Table 2:** Grading of the immune-staining for Grp94

Patients	negative (−)	moderate (1 +)	strong (2 +)
Total (27)	1	4	22
Males/Females	0/1	2/2	10/11
Tumor staging			
I/II	1	2	7
III/IV	0	2	15
Tumor grading			
G2	1	1	12
G3	−	3	10

Scores for the intensity of the immunostaining for Grp94 was assessed by three independent observers who analyzed slices of the tumor sections on different occasions, as specified in Methods. Final score was assigned after the general agreement on the evaluation of any single sample. Only one negative score (female patient, CRC, stage I/II) was assigned in 27 samples. Thus, a statistically higher proportion of patients showed positivity for Grp94 (*p* = 4.172 × 10^−7^, exact binomial test). More tumors at later stages (*p* = 0.0074, exact binomial test) but not at higher grade (*p* = 0.34) stained strongly for Grp94. The Grp94 expression was neither associated with tumor stage (*p* = 0.39, Fisher's exact test) nor with tumor grading (*p* = 0.45).

### Grp94 can be measured in plasma of cancer patients only in complexes with IgG

**Figure 2:**
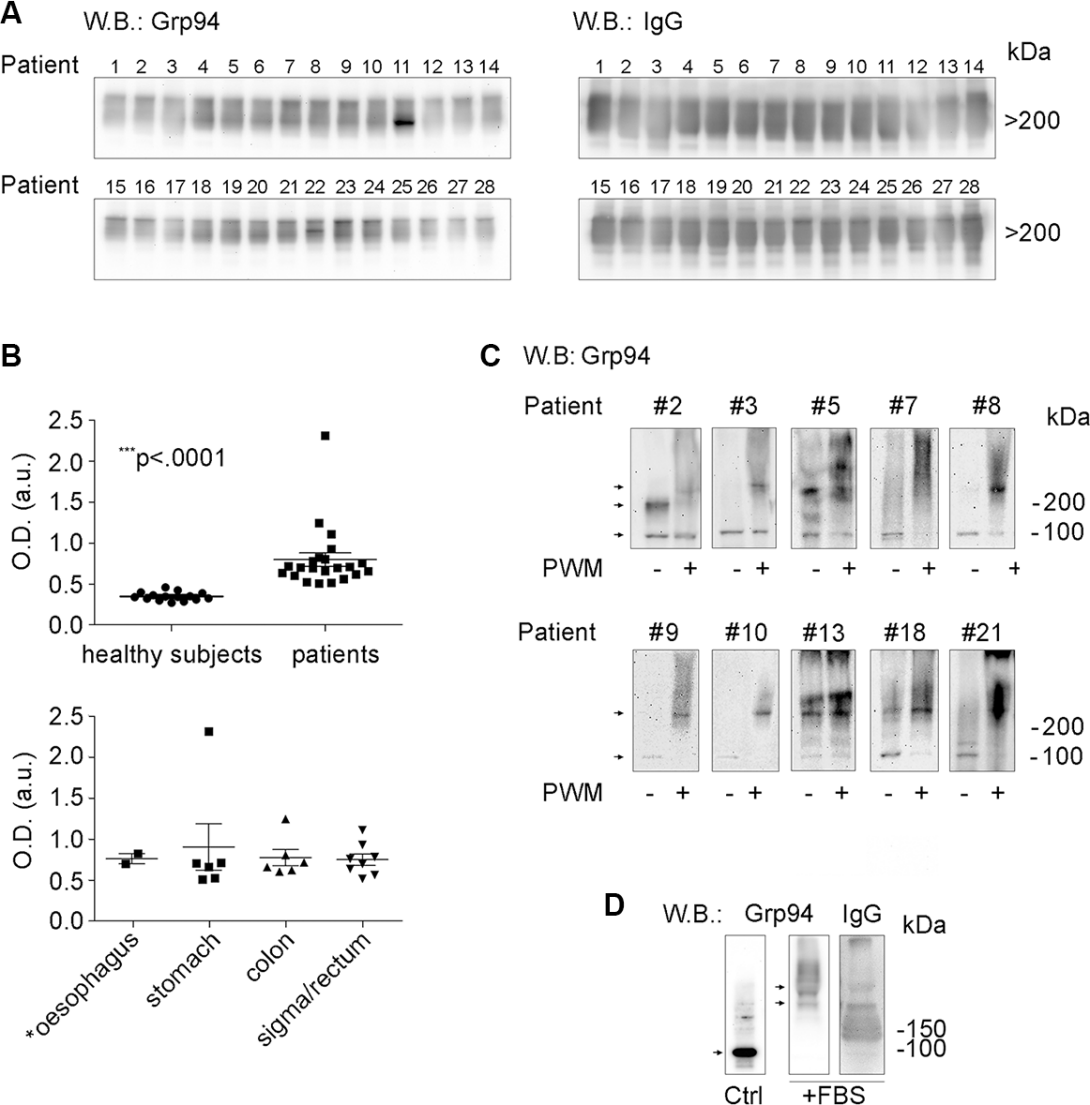
Grp94 in complexes with IgG is the form in which the tumor antigen Grp94 circulates in plasma and is presented by plasma cells (**A**) WB for Grp94 and IgG in plasma of all patients. Patients are numbered following the order by which they were analyzed. Ten μg of proteins of each plasma sample was loaded on to 4–20% polyacrylamide gel in denaturing conditions of PAGE, without boiling and reducing treatment of samples. WB was performed as specified in Methods, probing the membrane with primary anti-Grp94 monoclonal Abs (clone 9G10, Santa Cruz Biotech., Santa Cruz, CA, USA) followed by anti-rat HRP-conjugated secondary Abs, and with primary anti-human IgG polyclonal Abs followed by anti-sheep HRP-conjugated secondary Abs. Cropped WB of samples 1–14 and samples 15–28 originate from two distinct membranes. Patient #16 was then excluded from the following analyses for having a tumor that secondarily involved the large bowel. Only the Grp94-positive bands in the membranes are shown since no other band was visualized. Grp94 at masses higher than 200 kDa indicates the formation of complexes with IgG, as proved by the intense co-positivity for human IgG of the Grp94-positve bands (see WB on right). Specificity of the immune reaction was also evaluated by probing the membranes with only secondary Abs. (**B**) In the upper panel, scatter plots are shown of ELISA determinations of Grp94-IgG complexes in plasma of 15 healthy subjects and 22 cancer patients (13 males, 9 females). Measurements were made by testing each plasma sample in duplicate at the dilution of 1:128, and absorbance was measured at 450 nm (OD, optical density). Horizontal lines indicate the mean value with SE. Minimum and maximum OD values were, respectively, 0.27 and 0.458 in healthy subjects, and 0.506 and 2.31 in patients. The mean ± SE of OD values in healthy subjects was 0.346 ± 0.013, and in patients 0.796 ± 0.0817. Lower and upper 95% CI of the mean was 0.318 and 0.375 in healthy subjects, 0.627 and 0.966 in patients. The difference between the two groups was statistically significant (Mann-Whitney test, two-tailed). The lower panel shows the ELISA measurements in the same patients grouped by type of tumor. The mean ± SE OD values did not differ significantly among groups. The patient with the highest value of OD (2.31) died after two months from the admission. (**C**) Cropped WB for Grp94 on lysates of PBMCs of a representative number of patients (eight with large bowel adenocarcinomas and two with gastric carcinomas). Patients #3, #7, #9, #10 and #13 had tumors at stage III/IV. PBMCs were incubated in absence (control) and presence of the B cell stimulatory agent Pokeweed mitogen (PWM) at 20 μg/ml (final concentration), and after 10 days of incubation cells were lysed and treated as specified in Methods. The protein concentration was previously determined by means of calibration SDS-PAGE (10% polyacrylamide gel) and the same protein quantity (without boiling and reducing treatments) was loaded for each sample on to a 4–20% polyacrylamide gel and then probed with anti-Grp94 rat monoclonal Abs. Specificity of the reaction was assessed by probing the membrane with secondary Abs only. Stimulation by PWM leads to the increased expression of Grp94 at molecular masses much higher than the expected mass of the protein, consistent with the association of Grp94 in big complexes with IgG (WB for human IgG in Supplementary Figure S2B). Arrows mark the bands positive for Grp94 that in control PBMCs focus at molecular masses of both the monomer (100 kDa) and dimer (200 kDa), whereas in PWM-treated cells mostly appear at masses consistent with the formation of big complexes. Adjustments of the contrast and brightness were applied to each WB image. (**D**) Cropped WB for Grp94 and IgG on Grp94 in culture medium of PBMCs in absence (control, without incubation) and presence of 10% FBS (after incubation). Fifty μg of Grp94, in absence of reducing treatment, were loaded in each lane of a 4–20% polyacrylamide gel and probed with anti-Grp94 rat monoclonal Abs and anti-IgG sheep polyclonal Abs. Arrows mark the band of Grp94 that in absence of FBS appears in its free form, prevalently as monomer (100 kDa), whereas in the presence of FBS Grp94 focuses exclusively at high molecular masses in association with IgG. Blots for incubated Grp94 derive from a single membrane. No adjustments were made for the blots of Grp94, whereas slight adjustments for contrast and brightness were applied to the blot of IgG.

Since the marked positivity for Grp94 in cells of tumor infiltrates, especially B cells, supported the possibility that Grp94 could also be disseminated into the circulation, our next step was to measure Grp94 in patients’ plasma to see whether such measurement could be index of the tumor burden and diffusion, thus representing a useful biomarker. Previous works had stably demonstrated that when liberated in the extracellular milieu - as it also occurs in autoimmune diseases [27, 31] - Grp94 is never detected as a single protein, but is always found linked in big, stable complexes with IgG [26, 28]. To explore the possibility that Grp94-IgG complexes could also circulate in cancer patients, we first tested any single plasma sample with anti-Grp94 Abs in Western blotting (WB) (Figure 2A). While no immune reaction for Grp94 was detected in healthy control subjects (Supplementary Figure S2A), as also verified previously [28], Grp94 was instead present in the plasma of any patient with a variable degree of intensity, some patients showing an elevated burden of the protein (Figure 2A). Grp94 was always detected at molecular masses (> 200 kDa) consistent with the formation of big complexes, and in co-immunostaining with anti-human IgG Abs we verified that Grp94 was actually linked in complexes with IgG (Figure 2A).

Since WB does not permit to make any inference about the concentration of the protein detected, nor can it be used for diagnostic screening, we developed a sandwich ELISA to obtain a reliable measurement of Grp94 in plasma. The assay was based on the principle that after binding to circulating IgG to form stable non-immune complexes, Grp94 can still bind immune (anti-Grp94) IgG Abs. Indeed, it has been ascertained that binding of Grp94 to non-immune IgG occurs at sites other than the antigen-binding sites, involving a specific portion of the Grp94 molecule [30]. In our ELISA, anti-Grp94 (capture) Abs were used to detect antigenic sites of Grp94 bound to IgG, while anti-IgG (detection) Abs could reveal IgG linked to Grp94 in the complex. Positivity of the reaction could thus occur if Grp94 was present in the complex with IgG. By measuring the optical density of the immune reaction for circulating Grp94-IgG complexes, a highly significant increase was noted in plasma of patients with respect to healthy control subjects (Figure 2B). Of note is the finding that the highest values of optical density for Grp94-IgG complexes were found in patients whose plasma also showed the most intense immune reaction for Grp94 in WB, a result that supported the reliability of measurements made with the immunoassay. Apparently, among patients tested, there was no significant difference in the Grp94-IgG complex concentration as far as the site of tumor is concerned (Figure 2B, lower panel), but the highest values of optical density belonged to patients with tumors at the more advanced stages with the involvement of lymph nodes.

### Stimulation of B lymphocytes in PBMCs of cancer patients induces an increased expression of Grp94 in complexes with IgG

To further investigate the issue of the involvement of B lymphocytes as cells of tumor infiltrates mostly responsible for taking up Grp94 and presenting it in association with IgG, we set up experiments in which PBMCs of patients were cultured with the Pokeweed mitogen (PWM). PWM is used as specific stimulatory agent of B cell proliferation and Ig secretion, being able to induce the transformation of CD20^+^ cells into CD20^−^ Ab-secreting cells [32]. Thus, if B cells of patients actually encountered and captured Grp94 as tumor antigen, then, activation by PWM would also cause an increased expression of Grp94. After challenging PBMCs with PWM for ten days, cells were lysed and Grp94 detected in WB. To exclude that stimulation with the mitogen could induce an increase in the expression of other HSPs also identified as tumor antigens [15, 33] we measured the expression of both HSP90 and Grp78 in cell lysates. As shown in Figure 2 (panel C), incubation with PWM led to a marked rise in the Grp94 expression that, at variance with what occurred in the corresponding un-stimulated control in which Grp94 appeared at its expected molecular mass (100 kDa for the monomer, and 200 kDa for the dimer in non-reducing conditions), was found at higher molecular masses testifying the formation of big complexes (Figure 2C). That Grp94 was also linked to IgG in PWM-stimulated PBMCs was confirmed by WB for IgG in which bands positive for Grp94 also stained for IgG (Supplementary Figure S2B). No positivity was instead detected for either HSP90 or Grp78 in PBMCs stimulated with PWM (Supplementary Figure S2C), a result that confirmed the specificity of Grp94 as tumor antigen also present in B cells of cancer patients.

### PBMCs of cancer patients challenged with Grp94 show marked cellular differentiation into macrophages. Effects are mediated by Grp94 in complexes with IgG

**Figure 3:**
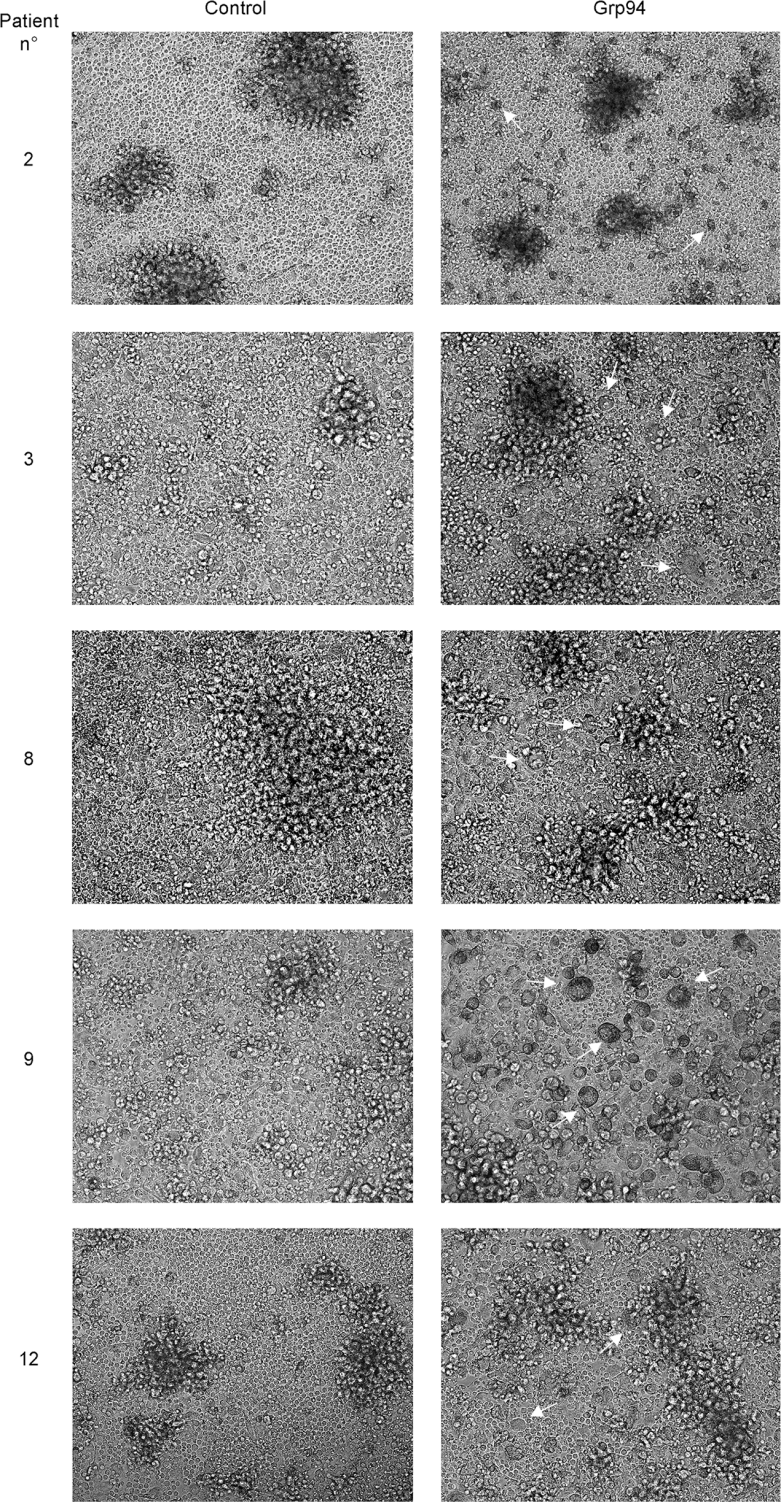
PBMCs of cancer patients challenged with Grp94 in complex with IgG are stimulated to differentiate into macrophages Microscopic evaluation of PBMCs of representative cancer patients (#2, #8 and #12, males; #3 and #9, females), with large bowel adenocarcinomas (patients #2, #3, #9 and #12) and gastric carcinoma (patient #8) cultured for ten days in both absence (control) and presence of 100 ng/ml of Grp94. Images are shown (20×) representative of several others taken at the optical microscope (Phase Contrast Leyca DMI 4000B, equipped with DFC camera 480). Arrows indicate the cells that mostly populate PBMC cultures after treatment with Grp94, identified as macrophages of various sizes.

If the soluble form of the tumor antigen was Grp94 in complexes with IgG, then, it would be expected that by challenging patients’ PBMCs with the antigen at proper concentrations could induce the activation of immune cells followed by an inflammatory response possibly directed against the cells presenting the antigen. To verify that Grp94 added to the culture medium of PBMCs was not present as free protein but only in complexes with IgG contained in FBS of the medium (see Methods), we set up preliminary experiments in which Grp94 at the highest concentration (100 ng/ml) was incubated with the culture medium in presence of 10% FBS, and positivity for Grp94 then tested in WB (Figure 2D). As expected, in absence of serum and without incubation, control Grp94 focused at its molecular mass of about 100 kDa, whereas after incubation with FBS, Grp94 was visible only at molecular weight that confirmed its stable binding with IgG (Figure 2D). It is also known that in absence of serum even a short incubation at 37°C causes the complete degradation of Grp94, a reason for which binding to IgG stabilizes and prolongs the activity of Grp94 in solution [30]. Thus, having proved that Grp94 in culture medium of PBMCs was present in complexes with IgG, we incubated PBMCs with Grp94 at the final concentrations of both 10 and 100 ng/ml, and analyzed the morphological characteristics of cells at the optical microscope. The entity of the inflammatory response was also evaluated by measuring the concentrations of a set of cytokines in the PBMC supernatant. After ten days incubation with Grp94, PBMCs showed peculiar morphological characteristics markedly different from the basal morphology of untreated PBMCs (Figure 3). Despite the expected inter-individual variability, mostly pertaining to quantitative aspects of the morphological changes, the common denominator of every picture in any patient was the appearance of numerous big cells with expanded cytoplasm and of heterogeneous shape, often clustered in agglomerates, suggestive of an accelerated rate of monocyte-to-macrophage differentiation, mostly evident at 100 ng/ml of Grp94. The quantitative analysis of the morphological transformation induced by Grp94 on PBMCs was evaluated by counting the number of big cells per field following the treatment compared to those in the corresponding, untreated control (Table 3). Although such a measurement was possible only in seventeen patients - the remaining ten patients shared a pattern of Grp94-induced big agglomerates of indistinct cells – a significantly higher number of transformed cells was found in Grp94-treated PBMCs, regardless of the gender of patients (Table 3).

**Table 3:** Number of cells identified as big monocytes/macrophages in both control and Grp94-treated PBMCs of cancer patients

patient	sex	average number of big cells/field	
		control	Grp94
# 2	M		5	17
# 3	F		4	10
# 4	F		13	21
# 7	F		4	10
# 8	M		1	8
# 9	F		6	19
# 10	M		4	10
# 11	M		4	16
# 12	M		0	8
# 14	F		3	11
# 16	M		5	7
# 17	M		1	11
# 20	F		5	15
# 23	M		3	9
# 24	F		9	14
# 25	M		14	17
# 27	F		10	26
sex ratio	9M/8F	median	4	11
		(range)	(0–14)	(7–26)

The cell count was measured in listed patients whereas in missing ten patients a pattern of big agglomerates was the common feature in Grp94-treated (100 ng/ml) PBMCs that completely obscured the visualization of individual cells. Numbers represent the average of cells counted in at least four fields of both control and Grp94-treated PBMC images taken at the optical microscope at least in duplicate for each sample, and analyzed using ImageJ 1.51a (NIH, USA). The median (range) of control cells in males and females was, respectively, 4 (0–14) and 5.5 (3–13), whereas following Grp94 treatment was 5.5 (3–13) and 14.5 (10–26) in males and females, respectively. No difference was found between males and females for the cell number in both control (Mann-Whitney test, *p* = 0.12) and Grp94-treated PBMCs (*p* = 0.1), whereas a highly significant difference was present for the addition of Grp94 to PBMCs in both all patients (*p* = 5.77 × 10^−5^) and males (*p* = 0.003) and females (*p* = 0.004) considered separately.

### Secretion of inflammatory cytokines (IL-6 and TNFα) from PBMCs of cancer patients is increased by Grp94, but the entity of the response is influenced by sex

**Figure 4:**
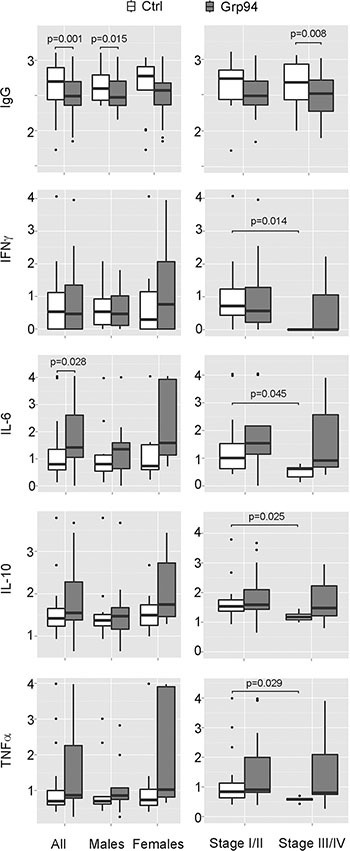
Grp94 in complex with IgG displays a sex-dependent stimulatory effect on cytokine secretion and a general significant inhibition of the IgG secretion from PBMCs of cancer patients Data of both IgG and cytokine concentrations measured in PBMC media in both absence and presence of Grp94 (100 ng/ml) are Log_10_ transformed and graphed in box plots with medians, first and third quartiles and outliers (when present). For the data of IFNγ, a conventional Log_10_ (1 + pg/ml) transformation was used to convert zero values of this cytokine into positive ones. Panels on left: data are graphed and analyzed for all patients and for the male and female group separately. Two patients (1 male and 1 female) were missing in the cytokine measurements. IgG values were normalized to any patient's B cell count. Differences in the concentration of each variable between control and Grp94-treated cells were assessed with the Mann-Whitney test (two-tailed) and the exact *p*-value for the statistical significance reported above the box plots. Panels on right: IgG and cytokine measurements were analyzed after grouping patients for stage of tumor. Differences in the concentration of any variable were calculated both in each group (basal *vs* treatment), and between groups (basal and treatment of stage I/II *vs* basal and treatment of stage III/IV). Significant differences with the exact *p*-value are reported.

It has been reported that when human monocytes are co-cultured with tumor cells from colorectal cancer patients they differentiate into macrophages that display an inflammatory, anti-tumor phenotype [34]. To test whether the appearance of numerous macrophages in the PBMC population following the challenge with Grp94 was consistent with the activation of an inflammatory response, we measured cytokines in cell media after two days incubation with Grp94. This end-point was useful to detect almost all the cytokines under investigation with the exception of IL-4 the concentration of which fell below the limit of detection in almost all patients, a fact that led to the exclusion of this cytokine in the following analyses. As shown in Figure 4 (left panels), in which effects on cytokine secretion by 100 ng/ml of Grp94 are graphed in box-plots, and in Supplementary Table S1, reporting the original data with both 10 and 100 ng/ml of Grp94, there was a concentration-dependent stimulatory effect of Grp94 on the secretion of inflammatory cytokines with the exception of IFNγ. Although IFNγ apparently did not change when all patients were analyzed together, in the sub-group of females there was a marked stimulation of IFNγ whose concentration rose from a median basal value of 1.39 pg/ml to 7.46 at 100 ng/ml Grp94 (Supplementary Table S1). IL-6 was the cytokine maximally stimulated by Grp94 especially 100 ng/ml, whereas increases in the concentration of other cytokines, though relevant, were not significantly different from basal values due to the large dispersion of values and the limited number of patients analyzed in each subgroup (Figure 4, left panels). However, it has to be noted that the increase in the cytokine secretion observed in all patients was almost exclusively sustained by female patients in whom the dose-dependent effect of Grp94 was more evident (Supplementary Table S1), a result that suggested a sex-dependent reactivity of PBMCs to the stimulation of Grp94.

When patients were grouped by tumor stage, the most evident result was the significant reduction of the basal secretion of any cytokine in patients with tumors at more advanced stages (III/IV) with respect to those with tumors at stage I/II (Figure 4, panels on right and Supplementary Table S2), to suggest that a defective immune response might occur as the disease progresses. Analyzing the Grp94 effect on cytokine secretion in these subgroups of patients, IL-6 turned out to be still the cytokine that underwent marked increases, though not statistically significant (Figure 4, panels on right, Supplementary Table S2).

### Grp94 significantly inhibits the secretion of IgG from PBMCs of cancer patients

It has been reported that a significant infiltration of B lymphocyte-derived Ig commonly occurs in pre-malignant and malignant stroma of several solid human cancers [35] and that cancer, but not normal cells express high levels of IgG that promote the proliferation and the diffusion of the tumor [36]. Both tumor and circulating complexes of Ig are associated with an increased tumor burden and poor prognosis [37], a reason for which targeting B cells for reducing the aberrant Ig production has been proposed as a useful therapeutic approach in different human tumors [35–37]. In the light of these observations and of the significant infiltration of B cells and Grp94-positive plasma cells found in tumor stroma of our patients, we wanted to explore the effect of Grp94 on IgG production by PBMCs. A concentration-dependent, statistically significant reduction of the IgG concentration in cell media was observed with Grp94 in all patients and also in males and females considered separately (Figure 4, panels on left and Supplementary Table S1). In particular, in male patients, Grp94 caused similar median reductions of 26% and 25% at 10 and 100 ng/ml, respectively, whereas in females a clearer concentration-dependent effect of Grp94 was apparent, with median reductions of 27% and 38% at 10 and 100 ng/ml of Grp94, respectively (Supplementary Table S1). Similar, concentration-dependent and significant reductions in IgG were observed with Grp94 when data were analyzed in patients grouped for stage of the tumor (Figure 4, panels on right and Supplementary Table S2).

To investigate whether there was any correlation among the variables measured, in particular whether the reduction of IgG induced by Grp94 in PBMCs was associated with the increase of a specific cytokine, in particular IL-6, we analyzed the partial correlations among the five variables using the Spearman's correlation index [38]. Considering the relatively small sample size and the fact that variables were expressed on different scales with several outlier values, we focused on values of partial correlations computed on the original data, fixing a reasonably high correlation value, equal or greater than 0.70. By applying this robust method, it turned out that both in the whole group of patients and even more in the subgroups of males and females there was not any significant correlation among the variables considered. Apparently thus, the effect of Grp94 to inhibit the IgG secretion was independent of the stimulatory effect on the cytokines.

## DISCUSSION

In this work we wanted to address some specific, still unmet questions of clinical relevance, related to the role of Grp94 as tumor antigen. Besides confirming that Grp94 is the tumor antigen shared by different histological types of tumors of the GI tract, we proved that it can be measured in plasma of cancer patients in complexes with IgG, serving as diagnostic tumor biomarker with the additional property to modulate the inflammatory response in circulating immune cells. Results of IHC analysis demonstrated that Grp94 was intensely expressed in any tumor tissue irrespective of the anatomical site of the tumor that included oesophagus, the gastro-oesophageal junction and the large bowel (colon, sigma and rectum) (Figure 1 and Table 2). Although the immune reaction for Grp94 did not show any significant association with both tumor stage and grading, a more intense Grp94 expression was associated with most advanced tumor stages (Table 2). These results were in keeping with several others observations pointing to Grp94 as the most common antigen protein in a large number of both solid and hematological tumors [4–7, 15, 39]. Among the proteins identified as reliable markers of cancer, including other HSPs such as Grp78 and HSP90 [15, 33], Grp94 appears to be the most reliable for predicting cancer invasiveness, recurrence and metastasis, being thus linked to a negative prognosis of the tumor [15, 17, 18, 40]. The role of Grp94 as pro-oncogenic chaperone has been demonstrated in both animal and human cancer cells [12, 14, 41] where translocation of Grp94 from the ER to cell membrane has been demonstrated to be a tumor-specific feature with implications for the regulation of tumor proteins involved in the transmission of oncogenic signal [12]. The cell surface appearance of Grp94 has important clinical consequences since Grp94 might thus become an easily accessible therapeutic target [8, 12]. Besides adding further evidence to these observations, with the demonstration that Grp94 often appeared in the apical portion of cancer cells, our results also revealed that Grp94 markedly stained cells of tumor infiltrates (Figure 1 and Supplementary Figure S1) strongly suggesting that the protein could be disseminated into the circulation. This finding was closely related to the question of whether and how Grp94 could be detected and measured as soluble tumor antigen, the only condition that permits to establish the validity of a protein as diagnostic and prognostic biomarker.

We were able to demonstrate that Grp94 actually circulates in the plasma of cancer patients in complexes with IgG, the exclusive form in which Grp94 has been found in circulation when it is liberated outside the cell in pathological conditions [26–28]. Western blot analysis showed that Grp94 was present in the plasma of any patient, with inter-individual variability in the expression to indicate that some patients had a higher level of the protein with respect to others (Figure 2A). However, the immune reaction for Grp94 always appeared at molecular masses consistent with the formation of big, SDS-resistant complexes that the specific immune reaction confirmed to be formed with IgG (Figure 2A), as also previously reported [26–28]. That Grp94 in complexes with IgG was the soluble tumor antigen in cancer patients was further confirmed by an enzyme-linked immune assay specifically developed to address the need to measure Grp94-IgG complexes in human plasma as diagnostic and prognostic biomarker. The measurements with this ELISA actually proved that Grp94-IgG complexes were statistically more elevated in cancer patients with respect to healthy control subjects (Figure 2B), showing in addition that the site of tumor did not influence significantly the value of Grp94-IgG complexes. Of particular interest was the observation that the highest value of Grp94-IgG complexes (largely exceeding the median value) was measured in a patient who died after only two months from the analysis, strongly suggesting that the massive burden of the protein present in the plasma of this patient was expression of the higher rate of proliferation and metastatic diffusion of cancer cells.

The analysis on cells of tumor infiltrates allowed us to shed further light on the mechanisms contributing to the diffusion of the tumor antigen into the circulation. We verified that the most part of cells infiltrating any tumor specimen were B cells (Figure 1B), a result in keeping with similar recent observations made by others in different types of solid tumors [37]. Recruitment of B cells in the tumor environment is consistent with the role of antigen-presenting cells played by B lymphocytes [42] that, by taking up and presenting tumor antigen(s) on the surface, actively contribute to spread the tumor [35, 37]. Moreover, B lymphocytes present at the site of tumor are responsible for the production of Abs developed against the antigen(s) exposed on the surface or liberated from cancer cells [43]. We showed that Grp94-positive cells in tumor infiltrates were mostly plasma cells (Figure 1B), to suggest that following the capture of Grp94, B cells were induced to differentiate into Ab-secreting cells, a condition that thus rendered possible the detection of the antigen in the IHC analysis. That Grp94 was expressed at higher level in Ig-secreting B cells was confirmed by the experiments in which we exposed patients’ PBMCs to the challenge with PWM used to specifically induce a proliferative response in B lymphocytes [32]. In this condition, commonly used as positive control for testing the IgG production in the PBMC population [44], the Grp94 expression not only rose significantly in PBMCs but was always found at elevated molecular masses (Figure 2C) also justified by the association of Grp94 with IgG (Supplementary Figure S2B). It is tempting to speculate that IgG Abs developed in Grp94-positive plasma cells might be either immune, i.e., directed against Grp94 itself, or non-immune, as those that were shown to form stable complexes with Grp94 at site other than the antigen-binding site [30]. The latter possibility seems more probable since immune complexes are reversible in nature and under denaturing conditions of SDS-PAGE they dissociate, whereas in PBMCs we never found Grp94 at the expected molecular mass of the protein.

It has been observed that IgG Abs that are often found increased in tumor compared to corresponding normal tissues instead of driving an anti-tumor effect turn out to be pathogenic, since they promote tumor growth and invasiveness, and are thus correlated with a poor tumor prognosis [35–37, 45, 46]. The experimental evidence accumulated so far indicates that B cells infiltrating tumors have a pro-tumorigenic role sustained by complexes that the pathogenic IgG can exert distally, a reason for which targeting B cells for reducing the IgG production has been considered a useful therapeutic target to halt tumor progression [35, 43]. Our finding showing that circulating Grp94-IgG complexes were markedly increased in cancer patients would thus be in accord with the proposed pathogenic role of these complexes as they not only are responsible for the antigen spreading, but per se also drive inflammatory angiogenic potential, as previously demonstrated on vascular cells [27].

**Figure 5:**
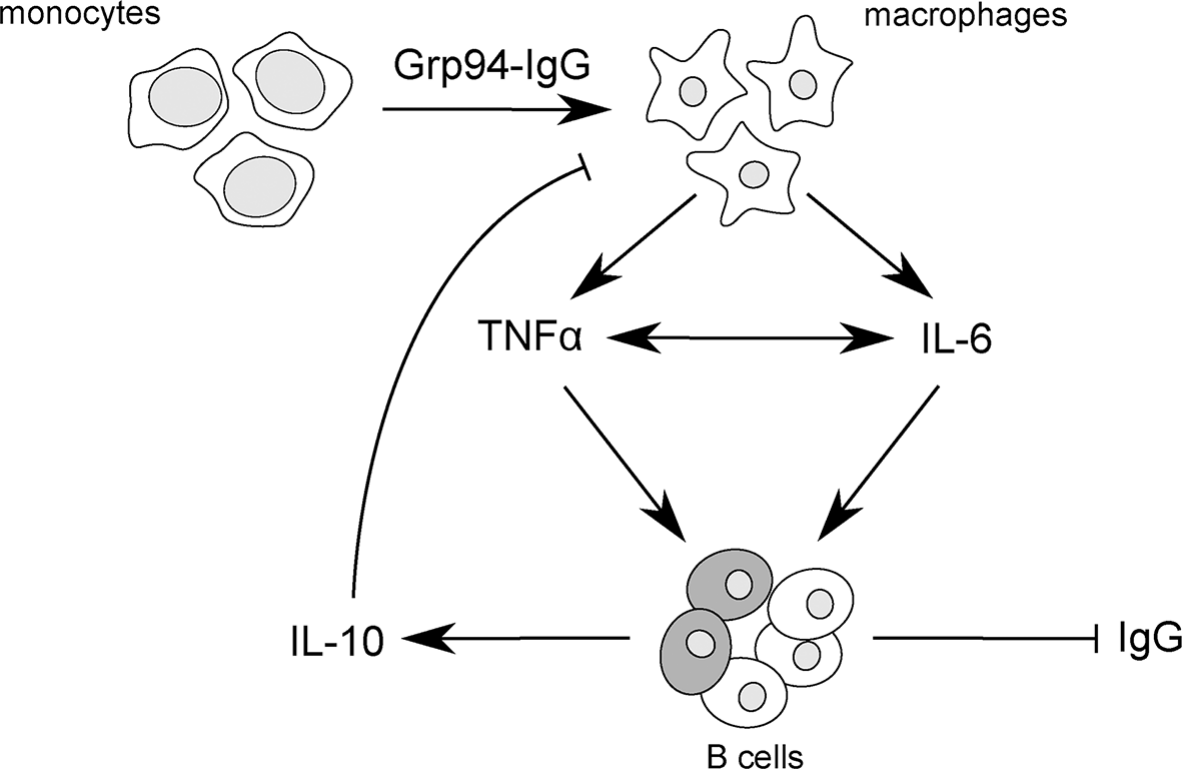
Scheme of proposed mechanism by which Grp94-IgG complexes might promote inflammatory responses in PBMCs of cancer patients Grp94-IgG complexes in cell culture medium of PBMCs drive the transformation of monocytes to macrophages that are induced to secrete both TNFα and IL-6. TNFα and IL-6 positively influence each other's secretion [50], potentiating the overall inflammatory effect. TNFα in turn acts as effective inhibitor of Ig production by B cells [54], in this way favoring the reactive production of IL-10 from a subset of regulatory B cells [55] (shadowed cells in the figure). IL-10 negatively regulates the macrophage production of both TNFα and IL-6, thus counteracting the immune response. In this view, the predicted mechanism of the antigen-driven inflammatory response coupled with the inhibition of IgG secretion would be mostly mediated by TNFα.

The identification of a specific tumor antigen is the prerequisite for developing a vaccine to use against that tumor, and a great deal of effort has been made to develop therapeutic vaccines, including those based on Grp94 as either subsidiary or alternative therapeutic option to the chemotherapeutic agents for the cure of different tumors [8, 20, 23, 24]. Based on this principle, and considering that our results indicated that Grp94 in complexes with IgG was the soluble form of the tumor antigen, we wanted to test the immune response in PBMCs of cancer patients challenged with Grp94, at both 10 and 100 ng/ml. Previous results indicated that irrespective of the nature of IgG, Grp94 can rapidly bind to, and form stable complexes with IgG, and we actually proved that this occurred with IgG of FBS of the culture medium (Figure 2D), so that effects measured on PBMCs after the addition of Grp94 could only be attributable to the complexes that it forms with IgG. Under this experimental condition, PBMCs underwent a series of morphological and functional modifications that in some aspects appeared to be also dependent on the gender of patients. Thus, whereas PBMCs of both male and female patients showed similar qualitative changes, characterized by the appearance of macrophages of different dimensions and shape often assembled in clusters (Figure 3), the response was markedly different as far as the cytokine secretion was concerned (Figure 4 and Supplementary Table S1). Only female patients were responsible for the rise in the concentration of the cytokines observed in all patients in the presence of Grp94, whereas in the group of males the value of any cytokine, with the only exception of IL-6, did not change with respect to the basal value (Figure 4). Apparently, thus, PBMCs of females displayed a particular sensitivity to the same Grp94 concentrations that in males instead caused little or no effects at all, and the cytokines that in female PBMCs underwent the highest stimulation were, in decreasing order of intensity, IL-6, IFNγ, TNFα and IL-10 (Supplementary Table S1). Since it is known that IL-10 exerts an inhibitory effect on the secretion of both IL-6 and TNFα from human monocytes [47, 48], the increase of IL-10 can only be interpreted as the response to the secretion of IL-6 and TNFα developed in order to counterbalance the inflammatory effect of these two cytokines. Macrophages are critical source of TNFα [49, 50] and, in particular, macrophages that infiltrate the tumor and circulate in colorectal cancer patients are identified as pro-inflammatory, being able to mediate tumor cell killing by the antigen-driven secretion of both IL-6 and TNFα [34, 49, 51]. Furthermore, the expression of immune cell-derived TNFα has recently been recognized as the most important predictor of the positive anti-tumor response attributed to TNFα in colorectal cancer [52]. Our results would thus be consistent with an antigen-driven activation of circulating macrophages that are induced to secrete inflammatory cytokines, especially IL-6 and TNFα, potentially associated with an anti-tumor effect. In line with this possibility is the potent inhibitory effect exerted by Grp94-IgG complexes on the IgG secretion (Figure 4 and Supplementary Table S1). This effect was stably significant regardless of whether the analysis was made on all patients or in patients grouped by sex or stage of tumor (Figure 4 and Supplementary Table S2). Although we cannot exclude that Grp94-IgG complexes might have a direct effect on B cells, other observations would rather favor the alternative possibility of an indirect, mostly TNFα-mediated mechanism. First, in a previous work we reported that Grp94, added to PBMCs in the same experimental conditions as the present ones, could significantly reduce the IgG secretion in human PBMCs only when Grp94 was the antigen expressed by immune cells, and this effect did not involve B lymphocytes directly [53]. Second, TNFα produced by macrophages is known to suppress the development of human B cells into Ig-producing cells with the consequent inhibition of Ig secretion [54]. In response to the secretion of inflammatory cytokines, a subset of B cells arises to produce IL-10 in order to counteract inflammation and keep the immune system in check [55]. Thus, the mechanism by which Grp94-IgG complexes might activate the inflammatory, anti-tumor response in PBMCs of cancer patients would predict that activated macrophages secrete both IL-6 and TNFα that, besides mutually reinforcing their own secretion [50], affect B lymphocytes, causing both the inhibition of the IgG production and the reactive anti-inflammatory secretion of IL-10 (Figure 5).

In conclusion, our results besides adding further evidence in support of Grp94 as the shared tumor antigen in tumors of the GI tract, prove that it can be measured in plasma as valuable diagnostic marker of disease in the form of complexes with IgG that also exert immune-modulating activities on circulating immune cells. Although further studies are necessary to corroborate our results, extending the investigation to other types of tumors and involving a larger number of patients, our study nevertheless clearly indicates that Grp94 in complexes with IgG can both stimulate the secretion of specific inflammatory cytokines and mostly inhibit the production of IgG in PBMCs of cancer patients, both these effects having predictable positive implications for halting the tumor growth *in vivo*.

## MATERIALS AND METHODS

### Study design and patients’ characteristics

A prospective study was conducted on 28 patients admitted to the Division of General Surgery (Civil Hospital of Padua) between 2011 and 2013 for surgical resection of primary tumors of the gastro-enteric tract (oesophageal, gastric and colorectal carcinomas). The criteria for eligibility were that patients did not receive any previous, specific antitumor treatment, nor that were they taking drugs that potentially could affect the immune system. No restriction was given for other variables (age, sex). Of each patient the laboratory and clinical data were collected together with radiological exams performed before the surgical intervention. A patient was excluded in the course of the study for having a tumor (leio-myosarcoma) that secondarily involved the large bowel. A formalin-fixed and paraffin-embedded block of each of the 27 carcinomas was cut into 4 μm sections stained with hematoxylin-and-eosin (H&E) to confirm histological diagnosis and evaluate other microscopic characteristics. Tumor stage was assessed according to the UICC TNM classification for the extent of tumor spread [56] and tumor grading of CRC based on the WHO classification (2009). Histological sub-typing of gastric cancer was performed according to Lauren's classification. Tumor size, depth of invasion, lymphatic and venous invasion were also determined.

Patients gave their informed written consent for having a blood sample drawn before surgical intervention and for the analysis of a specimen of the tumor tissue following tumor resection. A written informed consent for drawing a 10-ml blood specimen was also obtained from fifteen healthy subjects taken as control for ELISA measurements. The study was conducted according to the principles expressed in the Declaration of Helsinki and the study protocol was approved by the Ethics Committee (Comitato Etico per la Sperimentazione, Regione Veneto prot. N°1858).

### Immuno-histochemistry (IHC)

Three-μm sections of the paraffin-embedded block of each tumor were performed using a fully automated system (Bond^™^-Max, Leica, Newcastle Upon Tyne, UK). Sections were de-waxed and rehydrated and pre-treated using heat mediated antigen retrieval with sodium citrate buffer (pH6, epitope retrieval solution 1, Leica) for 30 minutes. Specimens were then incubated with rat monoclonal anti-Grp94 Abs (clone 9G10, Santa Cruz Biotechnology Inc. CA, 1:70 dilutions) for 30 min and a HRP-conjugated compact Bond Polymer Refine Detection system was then used according to the manufacturer's protocol. Staining was visualized with 3,3′- diaminobenzidine (DAB).

Immune-staining for Grp94 was graded for intensity based on a semi-quantitative score system: negative (−), moderate (1+), strong (2+). For each tumor specimen the adjacent portion of normal tissue served as its own negative control. Omission of primary Abs was also used as further negative control. In experiments in which double immunostaining was performed, the sections were also incubated with mouse monoclonal anti-human CD20cy Abs (clone L26; Dako, Glostrup, Denmark, 1:200 dilution) for 30 minutes and then labeled with the Bond Polymer Refine Red Detection Kit (Leica). The slides were then counterstained with Mayer's H&E. Each case was examined and scored blindly by three independent observers (MR, MC and PF) and the final score was assigned after general agreement. Images were acquired using Leica DMD108 Digital Microimaging Device and Software (Leica Microsystems, Milan, Italy).

### Preparation of Grp94 solutions

Grp94 was purified from the rat hepatocyte microsomal fraction through sequential chromatographic steps following the method of Lasa et al. [57] as also described previously [30]. The purified fractions of Grp94 were dialyzed in Slyde-A-Lyzer cassettes (3,500 kDa MWCO, Pierce, Rockford IL, USA) and proteins measured at 280 nm using the extinction coefficient of E_280_ = 0.884 for a 1-mg/ml solution and a path length of 1 cm [58]. Dialyzed Grp94 was stored at −20C° in 50-μl aliquots. Only the bands referred to Grp94, both monomer and dimer, were present in the purified fractions of Grp94 as assessed in SDS-PAGE and Western blotting (monoclonal anti-Grp94 Abs, Santa Cruz Biotechnology Inc., Santa Cruz CA, USA) [30]. Endotoxin contamination of the purified fractions of Grp94 was assessed in the QCL-1000 chromogenic LAL end-point assay showing that the endotoxin concentration was lower than the detection level (EU/ml < 0.125).

### Test for the Grp94-IgG complex formation

To ascertain that Grp94 added to the culture medium of PBMCs was not present as isolated protein but formed stable complexes with IgG present in FBS, Grp94 (100 ng/ml) was added to 4 ml of RPMI medium with 10% FBS (low IgG) and incubated at 37°C overnight. The solution was then dialyzed against milli-Q water on a 3.5 kDa MWCO membrane (SpectraPor, Spectrum Labs. CA, USA), lyophilized and re-suspended in 100 μl of Laemmli buffer for being processed in SDS-PAGE (4–20% poly-acrylamide gel) followed by Western blot analysis.

### Purification of peripheral blood mononuclear cells (PBMCs) from patients' blood

PBMCs were purified from the freshly drawn peripheral blood of patients using Ficoll–Paque density gradient according to the manufacturer's instructions (Amersham Pharmacia, Uppsala, Sweden). The blood sample was collected in EDTAcontaining Vacutainer tubes (Greiner Bio-one Company, Kremsmünster, Austria) the day before the surgery and immediately centrifuged at 1300 × g for 10 min to separate plasma from other cellular components. Plasma was frozen and stored at −80°C for further analysis, while PBMCs were purified as described [53].

PBMCs were plated at the concentration of 2 × 10^6^ cells/ml in a 24- or 48-well flat-bottomed plates with the medium (RPMI, Roswell Park Memorial Institute medium 1640, Euroclone Life Sciences Division, Milan, Italy), supplemented with 10% FBS (fetal bovine serum low IgG, Life Technologies, Gaithersburg, MD, USA), 1% L-glutamine (Sigma-Aldrich, St. Louis, MO, USA), and incubated at 37°C in a humidified 95% air and 5% CO_2_ atmosphere, in the absence (control) and presence of native Grp94 (10 and 100 ng/ml, final concentrations), in duplicate wells. Additional controls included cells treated with 100 μg/ml (final concentration) of puromycin (Sigma-Aldrich, St. Louis, MO, USA) to exclude any non-de novo synthesis of IgG, and 20 μg/ml (final concentration of) PWM (Sigma-Aldrich, St. Louis, MO, USA) used as positive control, i.e., as the mitogen of reference for inducing a polyclonal response in standardized test of functional activity of B cells [44]. After 3 day incubation, a 50-μl aliquot of supernatant of each sample was collected, centrifuged at 300 × g for 10 min, and immediately stored at −80°C for cytokine analysis. After 10 days, cells were harvested and centrifuged at 300 × g for 10 min, supernatants collected and stored at −80°C for further analysis whereas cells were treated as specified below for WB analysis. An aliquot of PBMCs just after their separation was also used for determining the percentage of CD19^+^lymphocytes in the flow cytofluorimetric analysis (anti-CD19 monoclonal Abs, Coulter Electronics FL, USA).

### Electrophoresis and Western blot analyses

SDS-PAGE and WB analysis were performed on both plasma samples and lysates of purified PBMCs. Plasma was obtained after centrifugation of blood sample of each patient as specified in the above chapter and analyzed on a 4–20% polyacrylamide gel followed by Western blot analysis with anti-Grp94 and anti-human IgG (Bethyl Laboratories Inc. TX, USA) Abs.

After incubation of 10 days, cell supernatant was removed, cells washed twice with 1 ml PBS and lysed with the Laemmli lysis buffer (50 mM Tris–HCl, pH 8.9, 5 mM EDTA, 380 mM glycine, 4% SDS, 10% glycerol) followed by boiling for 5 min. Cell lysates were analyzed in SDS-PAGE (both 10% and 4–20% polyacrylamide gel) and probed in Western blotting analysis with both anti-Grp94 and anti-human IgG Abs. Immunodetection was obtained using biotin conjugate, affinity-purified IgG (Bethyl Laboratories Inc. TX, USA) coupled with HRP-conjugated streptavidin (KPL Inc., Gaithersburg, MD, USA). Secondary Abs alone served as controls for excluding any false positive reaction.

### ELISA determination

To measure plasma Grp94, we applied the procedure developed in our laboratory based on the assumption that Grp94 present in the circulation always forms stable complexes with IgG [26, 28]. Stability of circulating Grp94-IgG complexes is considered the requisite for detecting Grp94 in an enzyme-linked immunoassay. Specifically, in our sandwich ELISA, a 96-well plate (200 μl/well) was covered with anti-Grp94 Abs (rabbit polyclonal, Enzo Life Sciences) and, after overnight incubation at +4°C the plate was washed with PBS solution and blocked with 3% BSA in PBS. Plasma samples (100 μl) of both patients and control subjects were diluted 1:128 in PBS with 0.1% Tween and 0.5% BSA and added in duplicate in predesigned wells. After 2.0-h incubation at room temperature under agitation, the plate was washed (with PBS containing 0.05% Tween) and probed with HRP-conjugated polyclonal anti-human IgG Fab2 Abs (Bethyl Laboratories, Inc.), diluted 1:10,000 in PBS with 0.05% Tween. The plate was then washed with PBS with 0.05% Tween and 100 μl of the Chromogen substrate solution (tetramethyl-benzidine, TMB, and hydrogen peroxide in citric acid, KPL Inc., Gaithersburg, MD, USA) were added to the wells. After five min, the reaction was stopped with 100 μl of 0.3 M sulfuric acid. The intensity of the immune reaction was detected by measuring the absorbance at 450 nm in a Victor spectrophotometer (Wallac, Turku, Finland) and expressed as arbitrary units of optical density.

### Multiplexed cytokine analysis

The measurement of the cytokines IL-4, IL-6, IL-10, IFNγ and TNFα was determined in the supernatant of patients' PBMCs using the Human Cytokine/Chemokine Magnetic Bead Panel protocol from the Milliplex^®^ Map Kit (Billerica, MA, USA). Forty μl of un-diluted supernatant of any PBMC culture were collected after 3-day incubation and analyzed with the immunoassay, according to the manufacturer's instructions. Measurements were performed using the Bio-Plex MAGPIX Multiplex Reader (Bio-Rad Laboratories, Hercules, CA, USA). The concentration of any cytokine was calculated on its own calibration curve and values expressed as pg/ml.

### Measurement of IgG in PBMC media

The IgG concentration in cell media was measured with an ELISA kit (ICL Labs. Portland, OR, USA). Media collected after 10 days incubation were diluted (1:10 and 1:50 dilutions) with the diluent running buffer. An aliquot of 100 μl was put in duplicate wells of a 96-well plate and incubated at room temperature for 2 h. After four washing steps with 200 μl of the washing solution, 100 μl of anti-human, HRP-conjugated IgG were added to the wells and left in incubation for 20 min at room temperature in the dark. A hundred μl of the chromogen-substrate solution (TMB and hydrogen peroxide in citric acid buffer, pH 3.3) were added after further washing, and the plate incubated at room temperature in the dark. After 10 min, the reaction was stopped with the addition of 100 μl of the stop solution (0.3 M sulfuric acid) and intensity of the reaction measured at 450 nm in a Victor spectrophotometer. The concentration of IgG in the supernatant was calculated on the calibration curve obtained with serial dilutions of human IgG standards (0–125 ng/ml), and expressed as ng/ml × 10^−5^ B cells, as determined with the flow cytofluorimetric analysis with anti-CD19 monoclonal Abs.

### Statistical analysis

Patients were categorized for age, gender and tumor stage and grade (Table 1). Values of cytokines that fell below the limit of detection by the instrument were treated as zero. Both the stage and grade of the tumor were considered as categorical variables. Since the values of cytokines were quite skewed with outliers, data are presented as medians (with range) and differences in concentrations of the variables measured at baseline and following treatments were assessed with the non-parametric test of Mann-Whitney, applying the powerful BH procedure to adjust the *p*-values for multiple comparisons [59]. All statistical tests were two-sided using α level of 0.05 as statistically significant. The analyses were conducted with the R software 3.1.3 [60].

## SUPPLEMENTARY MATERIALS TABLES FIGURES



## References

[1] Fu Z, Zhen H, Zou F, Wang X, Chen Y, Liu L (2014). Involvement of the Akt signaling pathway in ER-alpha36/GRP94-mediated signaling in gastric cancer. Oncology Lett.

[2] Fu Z, Deng H, Wang X, Yang X, Wang Z, Liu L (2013). Involvement of ER-alpha36 in the malignant growth of gastric carcinoma cells is associated with GRP94 overexpression. Histopathology.

[3] Zheng HC, Takahashi H, Li XH, Hara T, Masuda S, Guan YF, Takano Y (2008). Overexpression of GRP78 and GRP94 are markers for aggressive behavior and poor prognosis in gastric carcinomas. Hum Pathol.

[4] Langer R, Feith M, Siewert JR, Wester HJ, Hoefler H (2008). Expression and clinical significance of glucose regulated proteins GRP78 (BiP) and GRP94 (GP96) in human adenocarcinomas of the esophagus. BMC Cancer.

[5] Hu T, Xie N, Qin C, Wang J, You Y (2015). Glucose-regulated protein 94 is a novel glioma biomarker and promotes the aggressiveness of glioma via Wnt/beta-catenin signaling pathway. Tumour biology.

[6] Chhabra S, Jain S, Wallace C, Hong F, Liu B (2015). High expression of endoplasmic reticulum chaperone grp94 is a novel molecular hallmark of malignant plasma cells in multiple myeloma. J Hematol & Oncol.

[7] Boelens J, Jais JP, Vanhoecke B, Beck I, Van Melckebeke H, Philippe J, Bracke M, Jardin F, Briere J, Leroy K, Offner F, Lust S (2013). ER stress in diffuse large B cell lymphoma: GRP94 is a possible biomarker in germinal center versus activated B-cell type. Leukemia Res.

[8] Lee AS (2014). Glucose-regulated proteins in cancer: molecular mechanisms and therapeutic potential. Nat Rev Cancer.

[9] Wu S, Hong F, Gewirth D, Guo B, Liu B, Li Z (2012). The Molecular Chaperone gp96/GRP94 Interacts with Toll-like Receptors and Integrins via Its C-terminal Hydrophobic Domain. J Biol Chem.

[10] Melnick J, Dul JL, Argon Y (1994). Sequential interaction of the chaperones BiP and GRP94 with immunoglobulin chains in the endoplasmic reticulum. Nature.

[11] Nicchitta CV (2003). Re-evaluating the role of heat-shock protein-peptide interactions in tumour immunity. Nat Rev Immunol.

[12] Patel PD, Yan P, Seidler PM, Patel HJ, Sun W, Yang C, Que NS, Taldone T, Finotti P, Stephani RA, Gewirth DT, Chiosis G (2013). Paralog-selective Hsp90 inhibitors define tumor-specific regulation of HER2. Nat Chem Biol.

[13] Li X, Sun L, Hou J, Gui M, Ying J, Zhao H, Lv N, Meng S (2015). Cell membrane gp96 facilitates HER2 dimerization and serves as a novel target in breast cancer. Int J Cancer.

[14] Hou J, Li X, Li C, Sun L, Zhao Y, Zhao J, Meng S (2015). Plasma membrane gp96 enhances invasion and metastatic potential of liver cancer via regulation of uPAR. Mol Oncol.

[15] Kim SH, Ji JH, Park KT, Lee JH, Kang KW, Park JH, Hwang SW, Lee EH, Cho YJ, Jeong YY, Kim HC, Lee JD, Jang I (2015). High-level expression of Hsp90beta is associated with poor survival in resectable non-small-cell lung cancer patients. Histopathology.

[16] Huang CY, Batzorig U, Cheng WL, Huang MT, Chen WY, Wei PL, Chang YJ (2015). Glucose-regulated protein 94 mediates cancer progression via AKT and eNOS in hepatocellular carcinoma. Tumor Biol.

[17] Wang XP, Qiu FR, Liu GZ, Chen RF (2005). Correlation between clinicopathology and expression of heat shock protein 70 and glucose-regulated protein 94 in human colonic adenocarcinoma. World J Gastroenterol.

[18] Zheng HC, Takahashi H, Li XH, Hara T, Masuda S, Guan YF, Takano Y (2008). Overexpression of GRP78 and GRP94 are markers for aggressive behavior and poor prognosis in gastric carcinomas. Human Pathol.

[19] Wei PL, Huang CY, Tai CJ, Batzorig U, Cheng WL, Hunag MT, Chang YJ (2015). Glucose-regulated protein 94 mediates metastasis by CCT8 and the JNK pathway in hepatocellular carcinoma. Tumor Biol.

[20] Luo B, Lee AS (2013). The critical roles of endoplasmic reticulum chaperones and unfolded protein response in tumorigenesis and anticancer therapies. Oncogene.

[21] Usmani SZ, Bona RD, Chiosis G, Li Z (2010). The anti-myeloma activity of a novel purine scaffold HSP90 inhibitor PU-H71 is via inhibition of both HSP90A and HSP90B1. J Hematol & Oncol.

[22] Wang XY, Sun X, Chen X, Facciponte J, Repasky EA, Kane J, Subjeck JR (2010). Superior antitumor response induced by large stress protein chaperoned protein antigen compared with peptide antigen. J Immunol.

[23] Parmiani G, Testori A, Maio M, Castelli C, Rivoltini L, Pilla L, Belli F, Mazzaferro V, Coppa J, Patuzzo R, Sertoli MR, Hoos A, Srivastava PK (2004). Heat shock proteins and their use as anticancer vaccines. Clin Cancer Res.

[24] Schreiber TH, Deyev VV, Rosenblatt JD, Podack ER (2009). Tumor-induced suppression of CTL expansion and subjugation by gp96-Ig vaccination. Cancer Res.

[25] Angell H, Galon J (2013). From the immune contexture to the Immunoscore: the role of prognostic and predictive immune markers in cancer. Curr Opin Immunol.

[26] Pagetta A, Tramentozzi E, Corbetti L, Frasson M, Brunati AM, Finotti P (2007). Characterization of immune complexes of idiotypic catalytic and anti-idiotypic inhibitory antibodies in plasma of type 1 diabetic subjects. Mol Immunol.

[27] Tramentozzi E, Pagetta A, Frasson M, Brunati AM, Montopoli M, Finotti P (2009). Angiogenic transforming capacity of IgG purified from plasma of type 1 diabetic patients. J Cell Mol Med.

[28] Roveri A, Zaccarin M, Pagetta A, Tramentozzi E, Finotti P (2015). Proteomic Investigation on Grp94-IgG Complexes Circulating in Plasma of Type 1 Diabetic Subjects. J Diabetes Res.

[29] Tramentozzi E, Montopoli M, Orso G, Pagetta A, Caparrotta L, Frasson M, Brunati AM, Finotti P (2008). Stable complexes formed by Grp94 with human IgG promoting angiogenic differentiation of HUVECs by a cytokine-like mechanism. Mol Immunol.

[30] Pagetta A, Tramentozzi E, Tibaldi E, Cendron L, Zanotti G, Brunati AM, Vitadello M, Gorza L, Finotti P (2014). Structural insights into complexes of glucose-regulated Protein94 (Grp94) with human immunoglobulin G. relevance for Grp94-IgG complexes that form in vivo in pathological conditions. PloS One.

[31] Weber CK, Haslbeck M, Englbrecht M, Sehnert B, Mielenz D, Graef D, Distler JH, Mueller RB, Burkhardt H, Schett G, Voll RE, Furnrohr BG (2010). Antibodies to the endoplasmic reticulum-resident chaperones calnexin, BiP and Grp94 in patients with rheumatoid arthritis and systemic lupus erythematosus. Rheumatology.

[32] Bekeredjian-Ding I, Foermer S, Kirschning CJ, Parcina M, Heeg K (2012). Poke weed mitogen requires Toll-like receptor ligands for proliferative activity in human and murine B lymphocytes. PloS One.

[33] Bai Z, Ye Y, Liang B, Xu F, Zhang H, Zhang Y, Peng J, Shen D, Cui Z, Zhang Z, Wang S (2011). Proteomics-based identification of a group of apoptosis-related proteins and biomarkers in gastric cancer. Int J Oncol.

[34] Ong SM, Tan YC, Beretta O, Jiang D, Yeap WH, Tai JJ, Wong WC, Yang H, Schwarz H, Lim KH, Koh PK, Ling KL, Wong SC (2012). Macrophages in human colorectal cancer are pro-inflammatory and prime T cells towards an anti-tumour type-1 inflammatory response. Eur J Immunol.

[35] Andreu P, Johansson M, Affara NI, Pucci F, Tan T, Junankar S, Korets L, Lam J, Tawfik D, DeNardo DG, Naldini L, de Visser KE, De Palma M (2010). FcRgamma activation regulates inflammation-associated squamous carcinogenesis. Cancer Cell.

[36] Liao Q, Liu W, Liu Y, Wang F, Wang C, Zhang J, Chu M, Jiang D, Xiao L, Shao W, Sheng Z, Tao X, Huo L (2015). Aberrant high expression of immunoglobulin G in epithelial stem/progenitor-like cells contributes to tumor initiation and metastasis. Oncotarget.

[37] Affara NI, Ruffell B, Medler TR, Gunderson AJ, Johansson M, Bornstein S, Bergsland E, Steinhoff M, Li Y, Gong Q, Ma Y, Wiesen JF, Wong MH (2014). B cells regulate macrophage phenotype and response to chemotherapy in squamous carcinomas. Cancer Cell.

[38] Gibbons JD, Chakraborti S (2011). Chapter 11 in: Nonparametric Statistical Inference.

[39] Dejeans N, Glorieux C, Guenin S, Beck R, Sid B, Rousseau R, Bisig B, Delvenne P, Buc Calderon P, Verrax J (2012). Overexpression of GRP94 in breast cancer cells resistant to oxidative stress promotes high levels of cancer cell proliferation and migration: implications for tumor recurrence. Free Radical Biol & Med.

[40] Martinez-Aranda A, Hernandez V, Guney E, Muixi L, Foj R, Baixeras N, Cuadras D, Moreno V, Urruticoechea A, Gil M, Oliva B, Moreno F, Gonzalez-Suarez E (2015). FN14 and GRP94 expression are prognostic/predictive biomarkers of brain metastasis outcome that open up new therapeutic strategies. Oncotarget.

[41] Rachidi S, Sun S, Li Z (2015). Endoplasmic reticulum heat shock protein gp96/grp94 is a pro-oncogenic chaperone, not a tumor suppressor. Hepatology.

[42] Becker HJ, Kondo E, Shimabukuro-Vornhagen A, Theurich S, von Bergwelt-Baildon MS (2015). Processing and MHC class II presentation of exogenous soluble antigen involving a proteasome-dependent cytosolic pathway in CD40-activated B cells. Eur J Haematol.

[43] Gunderson AJ, Coussens LM (2013). B cells and their mediators as targets for therapy in solid tumors. Exp Cell Res.

[44] Hauser C, Wilhelm JA, Matter L, Schopfer K (1984). Spontaneous and pokeweed mitogen-induced in vitro IgG production specific for S. aureus cell wall determinants in man. Clin Exp Immunol.

[45] Qiu X, Zhu X, Zhang L, Mao Y, Zhang J, Hao P, Li G, Lv P, Li Z, Sun X, Wu L, Zheng J, Deng Y (2003). Human epithelial cancers secrete immunoglobulin g with unidentified specificity to promote growth and survival of tumor cells. Cancer Res.

[46] Zhang N, Deng H, Fan X, Gonzalez A, Zhang S, Brezski RJ, Choi BK, Rycyzyn M, Strohl W, Jordan R, An Z (2015). Dysfunctional Antibodies in the Tumor Microenvironment Associate with Impaired Anticancer Immunity. Clin Cancer Res.

[47] Chernoff AE, Granowitz EV, Shapiro L, Vannier E, Lonnemann G, Angel JB, Kennedy JS, Rabson AR, Wolff SM, Dinarello CA (1995). A randomized, controlled trial of IL-10 in humans. Inhibition of inflammatory cytokine production and immune responses. J Immunol.

[48] Rousset F, Garcia E, Defrance T, Peronne C, Vezzio N, Hsu DH, Kastelein R, Moore KW, Banchereau J (1992). Interleukin 10 is a potent growth and differentiation factor for activated human B lymphocytes. Proc Natl Acad Sci USA.

[49] Ruffell B, Coussens LM (2015). Macrophages and therapeutic resistance in cancer. Cancer Cell.

[50] Bonta IL, Ben-Efraim S (1993). Involvement of inflammatory mediators in macrophage antitumor activity. J Leukocyte Biol.

[51] Zhang QW, Liu L, Gong CY, Shi HS, Zeng YH, Wang XZ, Zhao YW, Wei YQ (2012). Prognostic significance of tumor-associated macrophages in solid tumor: a meta-analysis of the literature. PloS One.

[52] Reissfelder C, Stamova S, Gossmann C, Braun M, Bonertz A, Walliczek U, Grimm M, Rahbari NN, Koch M, Saadati M, Benner A, Buchler MW, Jager D (2015). Tumor-specific cytotoxic T lymphocyte activity determines colorectal cancer patient prognosis. J Clin Invest.

[53] Tramentozzi E, Zamarchi R, Pagetta A, Brunati AM, Rossi E, Tibaldi E, Finotti P (2011). Effects of glucose-regulated protein94 (Grp94) on Ig secretion from human blood mononuclear cells. Cell Stress & Chaperon.

[54] Kashiwa H, Wright SC, Bonavida B (1987). Regulation of B cell maturation and differentiation. I. Suppression of pokeweed mitogen-induced B cell differentiation by tumor necrosis factor (TNF). J Immunol.

[55] Rosser EC, Mauri C (2015). Regulatory B cells: origin, phenotype, and function. Immunity.

[56] Sobin LH, Gospodarowicz MK, Wittekind C (2009). TNM Classification of Malignant Tumours.

[57] Lasa M, Marin O, Pinna LA (1997). Rat liver Golgi apparatus contains a protein kinase similar to the casein kinase of lactating mammary gland. Eur J Biochem.

[58] Frey S, Leskovar A, Reinstein J, Buchner J (2007). The ATPase cycle of the endoplasmic chaperone Grp94. J Biol Chem.

[59] Benjamini Y, Hochberg Y (1995). Controlling the false discovery rate: A practical and powerful approach to multiple testing. J Royal Stat Soc. Series B (Methodology).

[60] R Core Team (2015). R: A Language and Environment for Statistical Computing. R Foundation for Statistical Computing.

