# Supplementary material for: Grp94 in complexes with IgG is a soluble diagnostic marker of gastrointestinal tumors and displays immune-stimulating activity on peripheral blood immune cells
Source: Oncotarget. 2016 Sep 20;7(45):72923–40. doi: 10.18632/oncotarget.12141 (PMC5341954; doi:10.18632/oncotarget.12141)
Supplement: Supplementary file 1 [file oncotarget-07-72923-s001.pdf]

Grp94 in complexes with IgG is a soluble diagnostic marker of gastrointestinal tumors and displays immune-stimulating activity on peripheral blood immune cells

Supplementary Materials

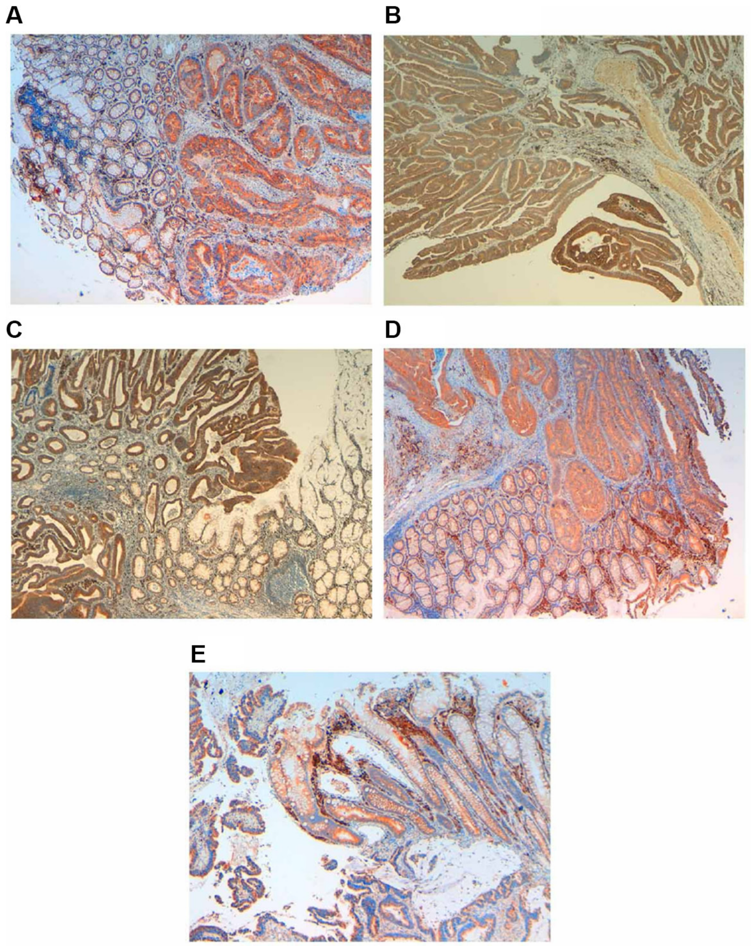

Supplementary Figure S1: Grp94 marks cells of tumor stroma. IHC for Grp94 in specimens of adeno-carcinomas of large bowel (**A–E**), showing the net separation of the Grp94-positive tumor from the normal, Grp94-negative tissue (A–D), and the intense staining for Grp94 in cells infiltrating the tumor stroma (A–E). In e (G2, stage I/II adenocarcinoma), a faint staining for Grp94 was apparent in the tumor tissue whereas stroma stained intensely. Magnification of 10×.

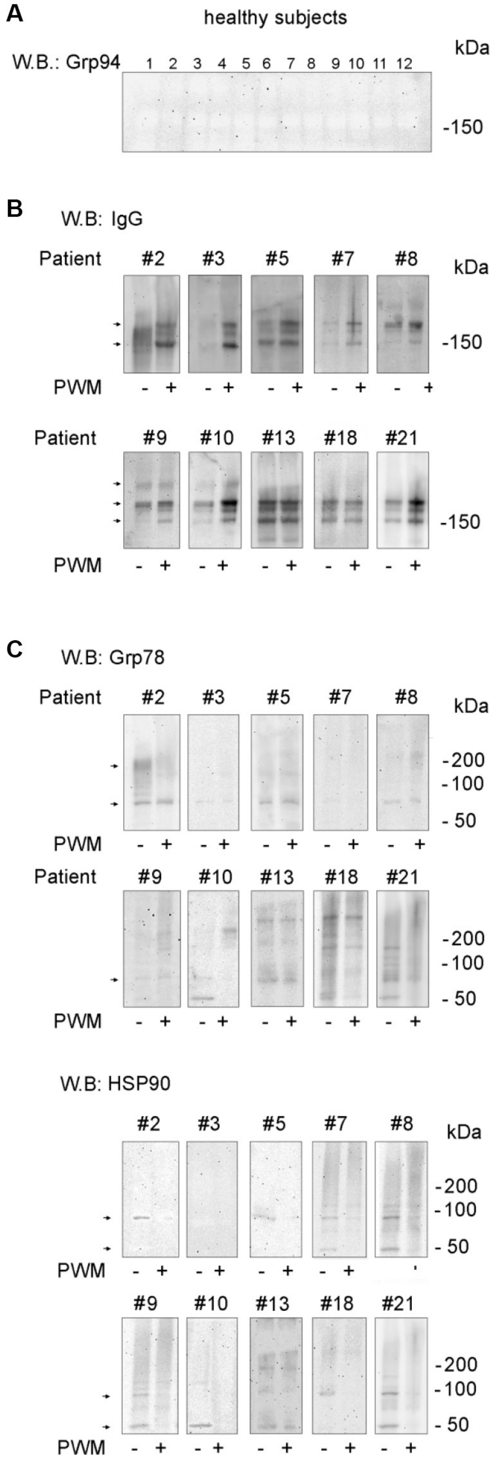

Supplementary Figure S2: Grp94 expression in PWM-stimulated PBMCs of cancer patients is associated with IgG. (**A**) Cropped WB for Grp94 in plasma of twelve representative healthy subjects. The same protein concentration as that used for patients was loaded in each lane of a 4–20% polyacrylamide gel and the membrane was probed with the same primary and secondary Abs as those reported in the legend to Figure 2A. The blotting derives from a single membrane and adjustment of both brightness and contrast was applied to render the frame of empty lanes visible. The negative bands at 150 kDa refers to the track of integer IgG, whereas no tracks of bands are shown at MW > 200 kDa that instead were positive for Grp94 in patients' plasma (Figure 2A). (**B**) Cropped WB for IgG on the same samples of PBMC lysates previously exposed to anti-Grp94 Abs (Figure 2C) to show that the Grp94-positive bands also co-stain for human IgG. Membranes were incubated with primary anti-IgG sheep polyclonal Abs, followed by incubation with anti-sheep HRP-conjugated Abs. Specificity of the reaction was assessed by probing the membrane with secondary Abs only. (**C**) The same samples as in B were also probed with primary anti-Grp78 and anti-HSP90 polyclonal Abs (Santa Cruz Biotechnology Inc. CA). Secondary Abs were anti-goat and anti-rabbit HRP-conjugated Abs for detecting immune reactions for Grp78 and HSP90, respectively. No specific positivity for both Grp78 and HSP90 is apparent following stimulation of PBMCs with PWM. Cropped WB are shown with adjustments of the contrast and brightness.

Supplementary Table S1: Dose-dependent effect of Grp94 on the inhibition of IgG and stimulation of cytokines from PBMCs of cancer patients grouped by sex

	Patients	Basal value	Grp94 (ng/ml)	
			10	100
IgG (ng/ml)	All	499.41 (53.2–1283.9)	424.44** (82.5–1095.2)	309.87** (70.4–1140.9)
	M	396.68 (226.6–1283.9)	292.4* (126.2–1074.4)	297.34* (143.2–1125.8)
	F	597.21 (53.2–1103.7)	436.2 (82.5–1095.2)	373.44 (70.4–1140.9)
IFNγ (pg/ml)	All	2.39 (0.0–11597.6)	2.79 (0.0–10836.7)	1.91 (0.0–9012.0)
	M	2.39 (0.0–119.2)	2.63 (0.0–102.6)	1.91 (0.0–63.3)
	F	1.39 (0.0–11597.6)	4.18 (0.0–10836.7)	7.46 (0.0–9012.0)
IL-6 (pg/ml)	All	6.29 (1.4–10995.4)	10.97 (1.2–11248.9)	26.15* (1.0–11361.2)
	M	6.36 (1.4–9431.2)	14.83 (1.2–11239.2)	22.46 (1.0–10120.6)
	F	5.46 (1.7–10995.4)	9.77 (1.5–11248.9)	41.24 (5.2–11361.2)
IL-10 (pg/ml)	All	26.26 (8.6–6172.8)	28.2 (5.4–4790.1)	35.36 (4.4–4708.1)
	M	23.57 (8.6–6172.8)	24.76 (5.4–4790.1)	29.61 (4.4–4708.1)
	F	31.40 (9.9–472.5)	38.47 (6.7–1295.7)	56.73 (19.4–2752.9)
TNFα (pg/ml)	All	5.0 (2.52–9757.1)	6.99 (1.7–10943.4)	7.34 (1.82–9528.1)
	M	5.0 (2.67–1021.4)	5.93 (3.2–893.3)	7.2 (1.82–664.9)
	F	5.57 (2.52–9757.1)	9.325 (1.7–10943.4)	11.27 (4.5–9528.1)

Original data are reported of any single measurement and expressed as medians with minimum and maximum in parenthesis. IgG and cytokines were measured in the supernatant of PBMCs in both absence (control) and presence of Grp94, as specified in Methods. IgG values are normalized to patients' B cell count. All patients were 27, males 14 and females 13. Two patients (1 male and 1 female) were missing in the cytokines measurements. Data obtained with 100 ng/ml of Grp94 were also reported in Figure 4 and graphed in box plots.

*$p < 0.02$, **$p < 0.001$ (Mann-Whitney test), comparisons made with the corresponding basal values.

Supplementary Table S2: Basal and Grp94-induced secretion of IgG and cytokines from PBMCs of patients grouped by tumor stage

	**Stage I/II (n = 10)**			**Stage III/IV (n = 17)**		
	**Basal value**	**Grp94 (ng/ml)**		**Basal value**	**Grp94 (ng/ml)**	
		**10**	**100**		**10**	**100**
IgG (ng/ml)	540.14 (53.2–1283.8)	424.4 (89.9–1095.8)	309.87 (70.4–1140.9)	478.1 (100.9–1151.3)	§364.3* (82.5–616.2)	§335.4** (79.6–1043.7)
IFNγ (pg/ml)	4.25 (0.0–11597.6)	5.13 (0.0–10836.7)	2.71 (0.0–9012.0)	¶0.00* (0.0–0.0)	0.625 (0.0–36.7)	0.00 (0.0–167.7)
IL-6 (pg/ml)	10.18 (2.7–10995.3)	10.97 (1.2–11248.9)	34.73 (1.0–11361.2)	¶4.25* (1.36–6.36)	16.1 (3.75–8823.3)	9.135 (2.51–8022.5)
IL-10 (pg/ml)	34.16 (8.56–6127.8)	28.2 (5.39–4790.1)	38.79 (4.41–4708.1)	¶14.93* (9.9–28.6)	26.48 (10.0–742.9)	30.24 (6.27–903.0)
TNFα (pg/ml)	6.86 (2.52–9757.1)	7.07 (1.7–10943.3)	8.00 (2.44–9528.1)	¶3.82* (2.69–5.0)	5.29 (3.69–2843.2)	6.31 (1.82–8018.4)

Original data for any measurement are reported and expressed as medians with minimum and maximum in parenthesis. Two patients (both with tumor stage III/IV) were missing in the cytokines measurements.

IgG and cytokines were measured in the supernatant of PBMCs in both absence (control) and presence of Grp94, as specified in Methods. IgG values are normalized to patients' B cell count. Data obtained with 100 ng/ml of Grp94 were also reported in Figure 4 as box plots.

$^{*}p < 0.04$, $^{**}p = 0.008$ (Mann-Whitney test with correction for multiple comparisons). ¶compared with basal values of stage I/II; §compared with the corresponding control basal value.
